# Supplementary material for: Evidence that a major subpopulation of fall armyworm found in the Western Hemisphere is rare or absent in Africa, which may limit the range of crops at risk of infestation
Source: PLoS One. 2019 Apr 4;14(4):e0208966. doi: 10.1371/journal.pone.0208966 (PMC6448938; doi:10.1371/journal.pone.0208966)
Supplement: S1 Table — (DOCX) [file pone.0208966.s001.docx]

S1 Table. Source information for fall armyworm specimens used in this study and reported in submitted manuscripts.

| Country | Region | Year | Collector |
| --- | --- | --- | --- |
| Chad | Logone Occidental | 2017 | N. A. Doyam |
| Chad | Mandoul | 2017 | N. A. Doyam |
| Central African Republic | Ombella-M'poko | 2017 | S. Ngarassem |
| South Africa | Limpopo | 2017 | H. Du Plessis |
| South Africa | North-West | 2017 | H. Du Plessis |
| Zambia | Serenje | 2017 | M. Rice |
| Ghana | Volta | 2016 | G. Goergen |
| Ghana | Greater Accra | 2016 | G. Goergen |
| Ghana | Ashanti | 2016 | G. Goergen |
| Togo | Lomé | 2017 | D. Koffi  K. Agboka  K. Tounou |
